# Complete mitochondrial genome of coconut hispine beetle *Brontispa longissima* (Coleoptera: Chrysomelidae: Cassidinae)

**DOI:** 10.1080/23802359.2019.1687341

**Published:** 2020-02-17

**Authors:** Rui Meng, Su Ao, Wei Xu, Baoqian Lv, Bo Cai

**Affiliations:** a Post-Entry Quarantine Station for Tropical Plant, Haikou Customs District, Haikou, Hainan, P. R. China;; b Hainan Province Engineering Research Center for Quarantine, Prevention and Control of Exotic Pests, Haikou, Hainan, P. R. China;; c Environment and Plant Protection Institute, China Academy of Tropical Agriculture Sciences, Haikou, Hainan, P. R. China;; d Key Laboratory of Integrated Pest Management on Tropical Crops, Ministry of Agriculture and Rural Affairs, Haikou, Hainan, P. R. China

**Keywords:** Cryptonychini, mitogenome, phylogenetic relationship

## Abstract

In the present study, we determined complete mitogenome of *Brontispa longissima*, the first for the tribe Cryptonychini. This mitochondrial genome contains 15,696 bp, with an A+T content of 72.2% (GenBank accession no. MN052901). All of the 22 tRNA genes displayed a typical clover-leaf structure, with the exception of tRNASer (TCT). 12 PCGs were initiated by ATN codons, and *ND1* started with TTG. Eight PCGs used the typical stop codon ‘TAA’ and ‘TGA’, while five PCGs terminated with incomplete stop codons "T". Phylogenetic analyses performed using the mitogenomes for the* B. longissima* and other seven species from subfamily Cassidinae.

The coconut hispine beetle, *Brontispa longissima* (Gestro) is a potential insect pest of palm cultivation worldwide (Nakamura et al. 2006). It is endemic to Indonesia and Papua New Guinea, and was accidentally introduced into several other Southeast Asian countries in the 20th century and spread rapidly after introducing to Hainan Province in China in 2002 (Lv et al. 2012). To date, no mitogenome has been studied for the whole tribe of Cryptonychini.

In this study, the complete mitochondrial genome of *B. longissima* is sequenced. Adult samples were collected from Xixiu Beach Park in Haikou (110°15′36″N, 20°1′48″E), Hainan, China. The specimens were deposited at −20°0 in the herbarium of Post-Entry Quarantine Station for Tropical Plant, Haikou Customs District, P. R. China (Specimen accession number IN06090101-0010–0015). The hind legs of a single individual were used for DNA extraction.

The complete circular mitogenome of *B. longissima* has a length of 15,696 bp (Genbank accession number MN052901). The nucleotide composition of *B. longissima* mitogenome was biased towards AT at 72.2%, and the total base composition was 39.2% A, 33.0% T, 9.9% G, and 17.9% C. The circular genome contained 13 protein-coding genes (PCGs), 22 tRNA genes, 2 rRNA genes, and an A + T rich region. The order and orientation of the above mitochondrial genes were identical to those of the ancestral insects (Boore 1999).

The length of 13 PCGs was 11,073 bp, 12 PCGs started with ATN, except that *ND1* initiated with TTG. Eight PCGs used the typical stop codon ‘TAA’ and ‘TAG’, while five PCGs terminated with incomplete stop codons ‘T’. All of the 22 tRNAs have a typical clover-leaf secondary structure, except for *trnS1* (*tRNASer* (TCT)) whose dihydrouridine arm formed a simple loop. All tRNAs had normal lengths, which varied from 61 to 71 bp. The 16S rRNA was 1277 bp long with an AT content of 78.6%, while the 12S rRNA was 729 bp long with an AT content of 75.2%. The non-coding region (putative control region) was 1241 bp in length with an A + T content of 75.4% and was well known for replication initiation.

The concatenated datasets of 13 PCGs in eight species representing six tribes of Cassidinae were used in phylogenetic analyses (Figure 1). Two species respectively from Chrysomelinae and Criocerinae were used as outgroups. Phylogenetic analyses were performed with Bayesian inference MrBayes 3.2.3 (Ronquist et al. 2012) and maximum-likelihood in RAxML 8.2.10 (Stamatakis 2014). The phylogenetic analysis showed that *B. longissima* of Cryptonychini has the closest relationship with *Callispa bowringii* of Callispini. Three species of *Cassida* belonging to Cassidini did not form monophyletic group. These results suggest that current mitogenome data remain limited in their ability to examine within-tribal relationships of this diverse subfamily.

**Figure 1.**
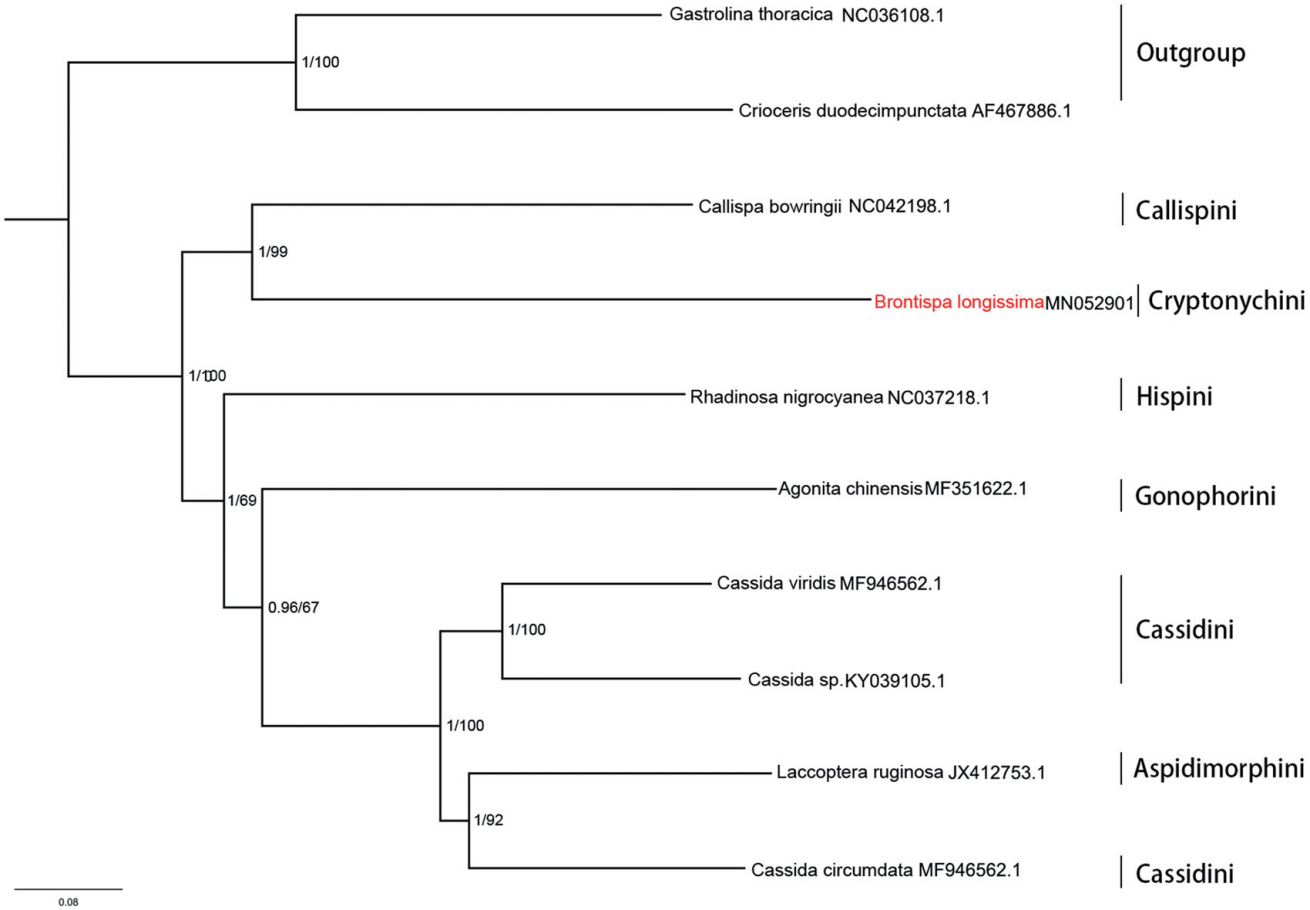
Mitochondrial phylogeny of *B. longissima* and other Cassidinae species. Numbers on branches are Bayesian posterior probabilities (left) and Bootstrap values (right).
